# Cigarette Smoke-Induced Pulmonary Inflammation Becomes Systemic by Circulating Extracellular Vesicles Containing Wnt5a and Inflammatory Cytokines

**DOI:** 10.3389/fimmu.2018.01724

**Published:** 2018-07-25

**Authors:** Diana Feller, Jozsef Kun, Istvan Ruzsics, Judit Rapp, Veronika Sarosi, Krisztian Kvell, Zsuzsanna Helyes, Judit E. Pongracz

**Affiliations:** ^1^Department of Pharmaceutical Biotechnology, School of Pharmacy, University of Pecs, Pecs, Hungary; ^2^Department of Pharmacology and Pharmacotherapy, Medical School, University of Pecs, Pecs, Hungary; ^3^Szentagothai Research Center, University of Pecs, Pecs, Hungary; ^4^Department of Internal Medicine, Clinical Centre and Medical School, University of Pecs, Pecs, Hungary

**Keywords:** chronic obstructive pulmonary disease, Wnt5a, extracellular vesicles, peroxisome proliferator-activated receptor gamma, inflammatory cytokines

## Abstract

Chronic obstructive pulmonary disease (COPD) is a devastating, irreversible pathology affecting millions of people worldwide. Clinical studies show that currently available therapies are insufficient, have no or little effect on elevated comorbidities and on systemic inflammation. To develop alternative therapeutic options, a better understanding of the molecular background of COPD is essential. In the present study, we show that non-canonical and pro-inflammatory Wnt5a is up-regulated by cigarette smoking with parallel up-regulation of pro-inflammatory cytokines in both mouse and human model systems. Wnt5a is not only a pro-inflammatory Wnt ligand but can also inhibit the anti-inflammatory peroxisome proliferator-activated receptor gamma transcription and affect M1/M2 macrophage polarization. Both Wnt5a and pro-inflammatory cytokines can be transported in lipid bilayer sealed extracellular vesicles that reach and deliver their contents to every organ measured in the blood of COPD patients, therefore, demonstrating a potential mechanism for the systemic nature of this crippling disease.

## Introduction

Chronic obstructive pulmonary disease (COPD) is an irreversible condition of airflow limitation characterized by inflammation and impaired respiratory gas exchange (1). The prevalence of COPD is approximately 10% among individuals older than 40 years of age (2). Furthermore, based on recent statistics, COPD is the seventh and fourth highest cause of disability and death worldwide, respectively (3). The main cause of COPD is tobacco smoking, but other factors, such as air pollution, occupational hazards, and infections can also initiate the disease (4, 5). Despite all the therapeutic efforts, COPD is not just irreversible but is also progressive in nature (6). Although COPD is recognized primarily as a lung disease, it has systemic manifestations and the comorbidities affect the heart, pancreas, skeletal muscle, and other organs (7). To suppress systemic inflammation, glucocorticoid therapy is used to treat acute exacerbations of the disease (8–10). Unfortunately, apart from the limited efficacy, unwanted side effects occur including histone deacetylase regulated resistance to corticosteroids (11, 12).

To find a permanent cure for COPD, more research is required into both drug target identification and disease pathomechanism. In recent years, peroxisome proliferator-activated receptor (PPAR)-gamma was identified as a central molecule in inflammation (13, 14). PPAR gamma, a member of the ligand-activated nuclear hormone receptor super family, controls gene transcriptions associated with lipid metabolism, adipogenesis, inflammation, and metabolic homeostasis (15, 16), making PPAR gamma an emerging anti-inflammatory and anti-oxidative target gene (13, 17). PPAR gamma agonists exert strong anti-atherogenic, anti-inflammatory (18–21), and anti-oxidant effects by inhibiting several inflammatory mediators (22–25).

Regulation of PPAR gamma, however, is essentially Wnt signaling dependent (26). Mostly, if the canonical or beta-catenin-dependent Wnt pathway is up-regulated, PPAR gamma is down-regulated (27–29). Additionally, PPAR gamma is also a non-canonical Wnt pathway target and is involved in the regulation of blood vessel growth *via* a non-canonical Wnt5a-dependent mechanism (30). Interestingly, Wnt5a is also a strong pro-inflammatory molecule that has been reported as a potent activator of cigarette smoke-induced inflammatory cytokine expression (30–32) and is characteristically up-regulated in squamous cell carcinomas (30). Cigarette smoke (CS) is also the initial trigger of macrophage activation (33) and macrophage polarization toward the classical M1 and the alternative M2 lineages (34). The anti-inflammatory activity of PPAR gamma is associated with inflammatory disorders of the lung leading to the hypothesis that treatment with PPAR gamma agonists can suppress inflammation (35, 36) and reverse CS-induced emphysema (37). Such theories have been substantiated by studies where PPAR gamma-deficient macrophages contributed toward spontaneous lung inflammation (38) and that PPAR gamma agonists promoted differentiation of the immune suppressive M2 macrophage phenotype (39). It is accepted that macrophage polarization is a multifactorial process and is associated with PPAR gamma in COPD. Based on the literature and our previous studies we hypothesized that although both canonical and non-canonical Wnt signaling regulate PPAR gamma, the non-canonical Wnt signaling pathway turns into dominant upon CS exposure. We also theorized that CS exposure induced local lung inflammation which becomes systemic *via* delivery of inflammatory messages in extracellular vesicles. To investigate our hypotheses, first, mice were exposed to CS. Then, mRNA and protein expressions of the most characteristic, pro-inflammatory Wnt ligand—Wnt5a—was measured. Next, expression of Wnt signaling, PPAR gamma and pro-inflammatory cytokines were investigated in aggregate cultures of primary human lung cell types and monocyte-derived macrophages. Finally, extracellular vesicles (EV-s) were isolated from sera of control volunteers and COPD patients to measure pro-inflammatory molecules.

## Materials and Methods

### Materials

Cigarette smoke extract (CSE) was diluted in cell culture media to a final concentration of 0.5%. Recombinant human Wnt5a (rhWnt5a) (Chinese Hamster Ovary Cell Line, CHO-derived Gln38-Lys380) protein was obtained from R&D Systems (Minneapolis, MN, USA) and used at a concentration of 1 µg/ml. PPAR gamma agonist, rosiglitazone (RSG), and antagonist GW9662 were purchased from Sigma-Aldrich (St. Louis, USA) and used at 10 µM concentrations each.

### Collection of Human Serum Samples

Human blood samples were collected from the Division of Pulmonology, 1st Department of Internal Medicine, Medical School, University of Pecs. A total of 5 COPD patients and 5 age-matched control samples were collected in blood collection tubes (BD Vacutainer, SST: BD SST™ Tubes with Silica Clot Activator and Polymer Gel, Franklin Lakes, NJ, USA). Blood samples were clotted for 30 min at room temperature. Samples were then centrifuged at 1,500 rpm for 10 min. Serum samples were stored at −80°C until used for further analysis.

### Animals

#### CS Exposure of Mouse Lungs

C57BL/6 male mice (20–25 g; *n* = 4–5/group) were housed in the animal facility of the Department of Pharmacology and Pharmacotherapy at the University of Pecs. Water and food was supplied *ad libitum*. Mice were exposed to CS in a whole-body smoke exposure system using 3R4F research cigarettes (College of Agriculture, University of Kentucky; Lexington, KY, USA). Passive smoking was performed in a manual smoking system (TE2; Teague Enterprises, Woodland, CA, USA). The exposures were performed 2 × 30 min/day with two cigarettes per occasion. The average concentration of the total suspended particles in the chamber was 70 mg/m^3^.

#### Microvesicle/Exosome Homing Study

BALB/C mice (20–25 g; *n* = 3/group) were housed in the animal facility of the Department of Immunology and Biotechnology at the University of Pecs. Water and food was supplied *ad libitum*. Microvesicles/exosomes were isolated from supernatants of Wnt5a-A549 cell lines and were fluorescently labeled with DiI (see below in detail). Native and fluorescently labeled microvesicles/exosomes were resuspended in 200 µl of phosphate-buffered saline (PBS) and injected into the tail veins of BALB/C mice. Control mice were injected with non-fluorescent exosomes.

### Mouse Lung Cell Isolation

Mice were anesthetized intraperitoneally with ketamine-xylazine (5 mg/kg), sacrificed by cervical dislocation, and their lungs were excised. Lung tissues were divided into three groups and were stored in RNA-later (Qiagen; Hilden, Germany) for molecular biological studies, in 1 ml lysis buffer for Western blot analysis or in 4% buffered formaldehyde (Szkarabeusz Ltd., Pecs, Hungary) for immunohistochemical examinations.

For the cell sorting procedure, 3 mice/group were anesthetized. Abdominal aorta was intersected, and mice were perfused with 10 ml of PBS through right ventricle to reduce blood content of the lung. To initiate the fine digestion 3 ml trypsin was applied, 10 ml PBS was used to wash out trypsin thereafter. 3 ml collagenase-dispase 3 mg/ml collagenase (Sigma-Aldrich, St. Louis, USA), 1 mg/ml dispase (Roche F. Hoffmann-LaRoche Ltd., Basel, Switzerland), and 1 u/μl DNase I (Sigma-Aldrich, St. Louis, USA) were used to fill up the lungs through the trachea. Pulmonary lobes were dissected into smaller pieces and digested in 10 ml collagenase-dispase for 50 min with continuous stirring. Digested lung cells were filtered with 70 µm cell-strainer (BD Becton, Dickinson and Company, Franklin Lakes, NJ, USA).

### Cell Sorting

Single cell suspensions of mouse lungs were labeled with anti-mouse CD45-FITC produced at the University of Pecs, Department of Immunology and Biotechnology and anti-mouse EpCAM1 (G8.8 anti-rat-PE) (40). Cell sorting was performed in a FACSAria III (BD Biosciences, San Jose, CA, USA) cell sorter. The following populations were collected: EpCAM^−^CD45^−^, EpCAM^+^CD45^−^, EpCAM^+^CD45^+^, and EpCAM^+^CD45^−^. The purity of sorting was above 99%.

### Preparation of CSE

Cigarette smoke extract solution was prepared using a modification of the method described earlier (41). CS of two 3R4F research grade cigarettes (Kentucky Tobacco Research and Development Center at the University of Kentucky, Lexington, KY, USA) were bubbled into 10 ml of RPMI without FBS (Lonza, Basel, Switzerland) using a Harvard peristaltic pump (Harvard Apparatus, P-1500, Holliston, USA) at a 550 ml/min flow rate for approximately 3 min. CSE solution was filtered through a 0.22 µm filter (TPP, Trasadingen, Switzerland). This solution was considered as 100% stock and diluted with culture media to 0.5% for further experiments. The pH of each CSE solution was measured as a mean pH 7.28. The prepared CSE solution was used within 30 min of preparation. The OD of the inner particles of the CSE was measured spectrophotometrically at the wavelength of 360 nm and compared to the control media.

### Cell Lines and Primary Cells

Human adenocarcinoma A549 cell line was obtained from American Type Culture Collection (Rockville, MD, USA). Transgenic Wnt5a overexpressing-A549 (Wnt5a-A549) cell line was created in our laboratory using lentiviral transgenesis (42). A549 cell lines were cultured in complete Dulbecco’s Modified Eagle Medium (DMEM) supplemented with 10% FBS. Primary human small airway epithelial cells (SAEC), normal human lung fibroblast cells (NHLF), and normal human lung microvascular endothelial cells (HMVEC-L) were cultured according to the manufacturers’ recommendations (Lonza, Basel, Switzerland). Cells were cultured in small airway epithelial cell growth medium (SAGM), fibroblast growth medium, and endothelial growth medium, respectively. All the above cell cultures were incubated at 37°C in humidified atmosphere containing 5% CO_2_.

### Isolation of Primary Human Monocytes

Macrophages were obtained from healthy blood donors (Hungarian National Blood Transfusion Service of Pecs, Pecs, Hungary). Peripheral blood mononuclear cells (PBMCs) were isolated from heparinized whole peripheral blood by standard density Ficoll-Paque (GE Healthcare, Sigma-Aldrich, St. Louis, USA) gradient centrifugation. Positive selection of monocytes was performed using CD14^+^ MACS colloidal superparamagnetic microbeads (Miltenyi Biotec, GmbH, Bergisch Gladbach, Germany) conjugated with monoclonal anti-human CD14 antibodies. Cell suspensions were mixed with the beads then CD14^+^ cells were separated by high gradient magnetic separation using MACS MultiStand, OctoMACS (Miltenyi Biotec GmbH, Bergisch Gladbach, Germany). Monocyte purity was regularly >85%. Macrophage differentiation was induced by incubation with 10 nM PMA, for 5 h, at 37°C in humidified atmosphere containing 5% CO_2_.

### Three-Dimensional (3D) Human Lung Aggregates

Three-dimensional lung aggregate tissues were generated as described in previous studies (30, 43, 44). Briefly, NHLF, SAEC, and HMVEC-L all purchased from Lonza (Lonza, Basel, Switzerland) were mixed (40% NHLF, 30% SAEC, and 30% HMVEC-L) in the absence or presence of peripheral monocyte-derived macrophages (30% NHLF, 25% SAEC, 25% HMVEC-L, and 20% macrophages) as described, then the cell suspensions were dispensed onto a low attachment 96-well U-bottom plate (Corning, New York, USA) and were centrifuged at 600 *g* for 10 min at room temperature. Tissue aggregates were maintained in 2:2:1 ratio of endothelial cell growth medium, SAGM, and fibroblasts growth medium. In the various experiments, aggregates were treated with 0.5% CSE or 1 µg/ml recombinant human Wnt5a (R&D Systems, Minneapolis, USA) for 48 h.

### RNA Isolation, cDNA Synthesis, and Quantitative RT-PCR

Total RNA was prepared from 3D cell cultures using NucleoSpin RNAII kit (Macherey-Nagel, Düren, Germany) with on-column DNase digestion. Random hexamer primers of the high capacity RNA to cDNA kit (Thermo Fisher Scientific, Waltham, MA, USA) was applied. For cDNA synthesis, 1 µg of total RNA was used according to the manufacturers’ protocol. For gene expression analysis, quantitative RT-PCR was performed using SensiFAST SYBR Green reagent (BioLine, London, UK) on ABI StepOne and StepOnePlus instruments (Thermo Fisher Scientific, Waltham, MA, USA). Data were analyzed using StepOne software and expression changes were normalized to beta-actin housekeeping gene.

### TaqMan Array

Total RNA was isolated from human monocytes then cDNA was synthesized. TaqMan master mix was combined with the cDNA samples then the mixed solutions were loaded onto the Human WNT Pathway, Fast 96-well TaqMan Array (Thermo Fisher Scientific, Waltham, MA, USA) plate. Gene expression was analyzed using ABI StepOnePlus instrument (Thermo Fisher Scientific, Waltham, MA, USA).

### Immunohistochemistry and Fluorescent Staining

Mice were anesthetized with ketamine-xylazine and their lungs were excised. After a formaldehyde fixation of the lung tissue, paraffin embedded sections were made using a microtome. Before proceeding with the staining protocol, the slides had to be deparaffinized by xylene and rehydrated. Antigen retrieval was performed in citrate buffer (10 mM citric acid, 0.05% Tween 20, pH 6.0). Fluorescent staining was performed using rat anti-human Wnt5a (1:100, Clone 442625, R&D Systems, Minneapolis, USA) monoclonal antibody as primary, then Alexa Fluor 488 donkey anti-rat IgG polyclonal antibody (1:200, Thermo Fisher Scientific, Waltham, MA, USA) as secondary antibodies.

The 3D human lung tissue aggregates were carefully removed from the 96-well plate and embedded into Cryomount (Histolab, Finland) embedding medium then immediately frozen to −80^o^C. Sections were made using a Leica CM1950 cryostat (Leica, Wetzlar, Germany). After a drying step, sections were fixed in cold acetone (Molar Chemicals Kft., Hungary) for 10 min. Fixed slides were blocked in PBS containing 5% BSA (Sigma Aldrich, St. Louis, USA) for 20 min. Wnt5a protein expression was detected by rat anti-human Wnt5a (1:100, Clone 442625, R&D Systems, Minneapolis, USA) monoclonal antibody as primary antibody. The secondary antibody was Alexa Fluor 488 donkey anti-rat IgG antibody (1:200, Thermo Fisher Scientific, Waltham, USA). Nuclei were counterstained with DAPI (1:1000, Serva, Heidelberg, Germany). Fluorescent images were captured using Olympus IX81 fluorescence microscope (Shinjuku, Tokyo, Japan) equipped with CCD camera and analysis software. Images were processed and analyzed with ImageJ.

### Flow Cytometric Bead Array (CBA) Cytokine Assay

Production of pro-inflammatory cytokines IL-6 and IL-8 were measured in cell culture medium using the multiplexed flow CBA Human Inflammatory Cytokines Kit (BD Biosciences, San Jose, CA, USA). For this assay, 50 µl culture medium/each sample of 3D aggregate cell cultures were collected at 48 h time points. The CBA kits were used according to the manufacturer’s instructions. The collected samples were diluted four times in the kit’s assay buffer. Diluted samples (50 µl) were added to equal amounts of fluorescent cytokine capture bead suspension (50 µl), and human inflammatory cytokine-phycoerythrin detection reagent (50 µl). Samples were then incubated for 3 h at room temperature. Labeled beads were measured using FACS Canto II flow cytometer (BD Immunocytometry Systems, Erembodegen, Belgium) with BD FACS DIVA software V6 and data were analyzed by FCS Express V3 software.

### EV Isolation From Human Serum Samples

Extracellular vesicles were isolated from human serum samples of 5 COPD patients and 5 healthy controls using the Total Exosome Isolation kit (from serum) (Invitrogen, Thermo Fisher Scientific, Waltham, MA, USA) following the manufacturer’s protocol. Frozen serum samples were thawed at room temperature. Samples were then centrifuged at 300 × *g* for 30 min at 4^o^C to remove cells and cell debris. Then the supernatant was centrifuged with 2,000 × *g* for 30 min at 4^o^C to collect megasomes/oncosomes in the pellet. The supernatant was then mixed with total exosome isolation reagent and vortexed. Samples were incubated at 2–8°C for 30 min, then centrifuged at 14,000 × *g* for 10 min at room temperature. The pellets containing EV-s (microvesicles/oncosomes) were collected and resuspended in cold EV resuspension buffer and stored at −80^o^C until further analysis.

### EV Isolation From A549 and Wnt5a-A549 Cell Lines

Extracellular vesicles were isolated from cell culture media following the protocol of the total exosome isolation (from cell culture media) kit (Invitrogen, Cat. number: 4478359). A549 and Wnt5a-A549 cell lines were cultured in DMEM supplemented with 10% FBS at 37°C in humidified atmosphere containing 5% CO_2_ until confluent. The medium was then removed, the cell layer washed in PBS, and FBS-free medium was added to the cultures. After 2 days at 37°C, 5% CO_2_, the FBS-free culture medium was harvested and centrifuged at 300 × *g* for 10 min at 4^o^C to remove cells and cell debris. Then the supernatant was centrifuged with 2,000 × *g* for 30 min at 4^o^C to collect megasomes/oncosomes in the pellet. Finally, total exosome isolation (for cell culture media) reagent was added in 0.5 volumes. Samples were incubated at 4°C overnight, and then centrifuged at 10,000 × *g* for 1 h at 4°C. The pellets containing EV-s were collected after centrifugation and resuspended in cold PBS until further analysis. Isolated EV-s were stored at −80°C.

### Staining of EV-s (Microvesicles or Exosomes)

Isolated microvesicles (exosomes) were centrifuged for 1 h at 4°C. Pellets were resuspended in PBS containing DiI (Molecular Probes, Thermo Fisher Scientific, Waltham, MA, USA) (dilution 1:10,000). Samples were incubated for 30 min at 37°C, then centrifuged with 10,000 × *g* for 1 h at 4°C. Supernatant was then removed and the pellet was resuspended in PBS.

### Fluorescence Analysis of Microvesicle (Exosome) Localization

Balb/c mice were injected with equal amount of stained or unstained exosome suspension in 200 µl of PBS. Mice were sacrificed after 20 h of exosome injection. Lung, liver, thymus, and the spleen were removed for fluorescence imaging of extracellular vesicle accumulations in mouse organs by the IVIS Lumina III *In Vivo* Imaging System using the Living Image Software (IVIS Imaging Systems) (PerkinElmer, Waltham, MA, USA). Fluorescence intensity was compared to fluorescence intensity of unstained control organs.

### Electron Microscopic Detection of Microvesicles (Exosomes)

Microvesicles isolated from cell lines were visualized by electron microscopy. First, microvesicles were resuspended in PBS and then fixed in 2.5% glutaraldehyde aqueous solution. 5 µl of fixed exosomes were embedded in the EM grid, which was flushed by water and blown dry for 24 h. Samples were treated with 5% uranyl-acetate at pH 7 for 5 min. The grid was examined using Morgagni 268D transmission electron microscope at 80 kV. Images were acquired using an integrated MegaView III digital camera (Olympus Soft Imaging Solutions GmbH; Munster, Germany).

### Sandwich Enzyme-Linked Immunosorbent Assay (ELISA)

Mega EV (oncosome) and micro EV (exosome) samples were resuspended in PBS containing 0.5% Tween 20. 100 µl of each sample was loaded to wells in the Aviva Systems Biology WNT5A ELISA Kit (Human) (OKEH00723). The plate was pre-coated with a Wnt5a-specific antibody (96-well plate, 12 × 8 well strips) and blocked. Standards were also provided by the manufacturer. Samples were incubated for 2 h at 37^o^C, then the wells were washed and incubated further with a biotinylated Wnt5a-specific detector antibody for further 1 h at 37^o^C. After a washing step, incubation continued with Avidin-HRP conjugate for 1 h at 37°C. The wells were then washed and TMB (3,3′,5,5′-Tetramethylbenzidine) substrate was added for 15 min at 37°C in the dark. The reaction was then stopped and the color reaction was measured at 450 nm absorbance. Results were calculated against the standard curve.

### Statistical Analysis

Statistical analysis was performed with GraphPad version 6 software. Data are presented as mean ± SEM, and statistical analysis was performed using the independent samples *t*-test and one-way ANOVA with Bonferroni correction. *p* < 0.05 was considered as significant.

## Results

### CS and CSE Trigger Wnt5a Up-Regulation in Both Mouse and Human Lung Tissues

**Figure 1:**
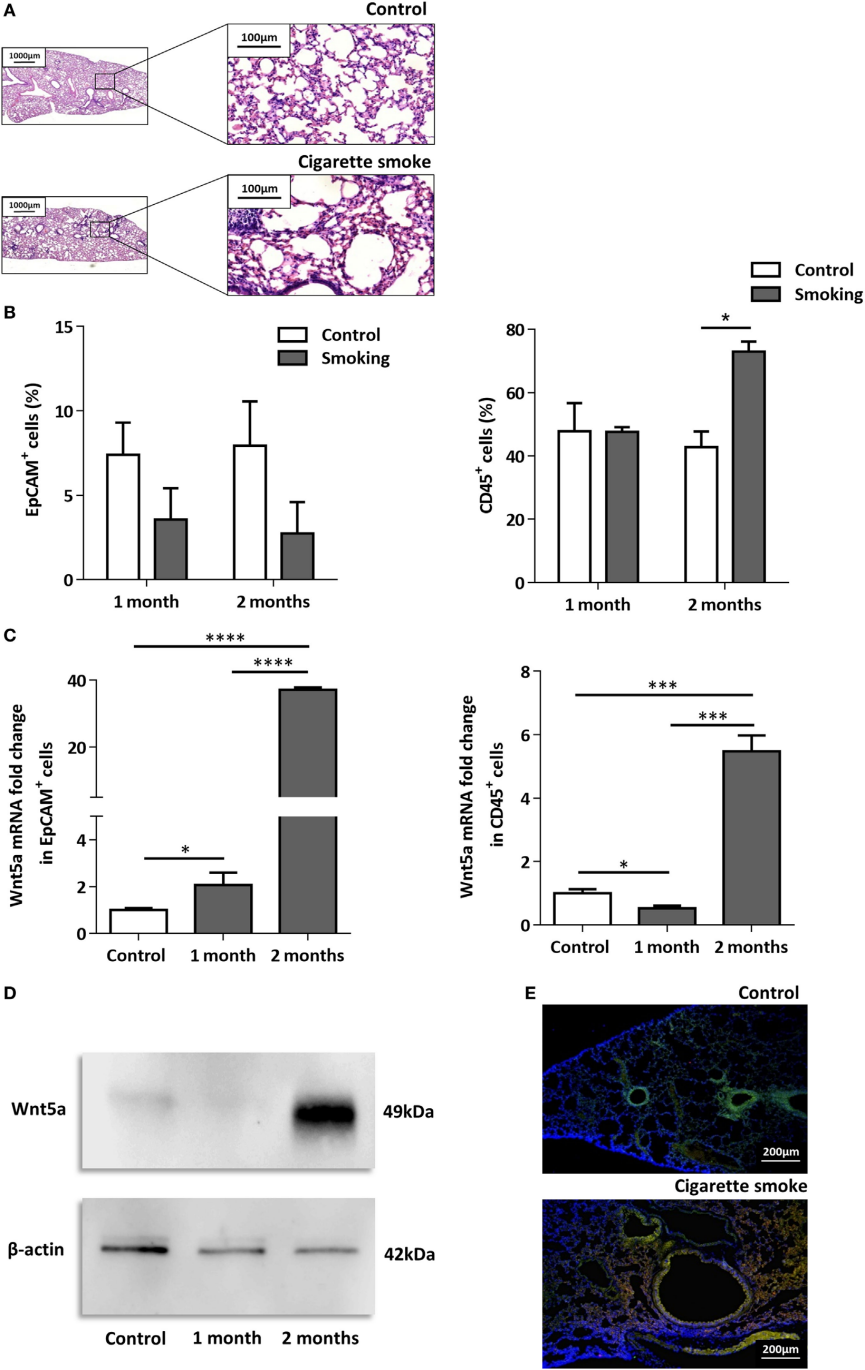
Effects of cigarette smoke (CS) on C57BL/6 mouse lungs. **(A)** Hematoxylin-eosin staining of paraffin embedded sections of healthy control and CS exposed lungs. Mice were exposed to CS for 1 h/day for 2 months. (Scale bars, 100 µm.) **(B)** Changes in EpCAM^+^ and CD45^+^ cell populations in control and CS exposed lungs determined by flow cytometry. **(C)** Wnt5a mRNA expression changes in sorted EpCAM^+^ and CD45^+^cells following 1 and 2 months of CS exposure (*n* = 3). **(D)** Western blot analysis of Wnt5a protein expression in control and CS exposed lung tissue extracts. **(E)** Detection of Wnt5a protein expression in control and CS exposed lung tissue sections [EpCAM^+^ (green)/Wnt5a (red)/Nucleus (blue), Scale bars, 200 µm].

**Figure 2:**
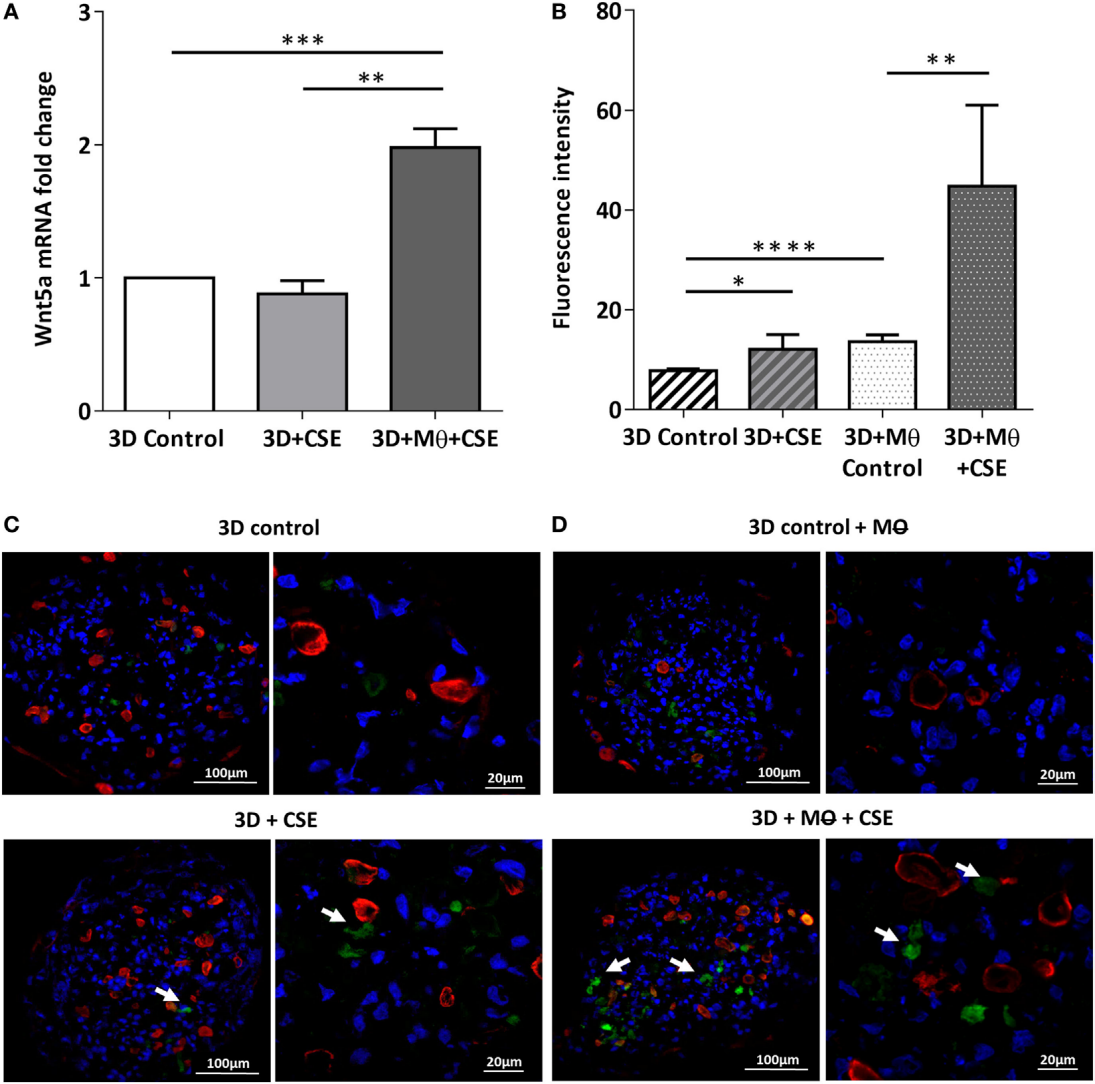
Effects of cigarette smoke extracts (CSE) on primary human 3D lung aggregate cultures. **(A)** Wnt5a mRNA was measured by qRT-PCR in control and CSE treated (48 h) aggregate cultures containing and not containing macrophages (M). **(B)** Fluorescence intensity of Wnt5a protein was analyzed by ImageJ. **(C)** Immunofluorescence staining of Wnt5a (green) and Cytokeratin7 (red) proteins in frozen sections of control aggregates and CSE (48 h) exposed aggregates not containing M. **(D)** Immunofluorescence staining of Wnt5a (green) and Cytokeratin7 (red) proteins in frozen sections of control aggregates and CSE (48 h) exposed aggregates containing M. Nuclei were stained by TO-PRO-3 Iodide (1:1,000, pseudo-blue). [Scale bars: 100 µm (magnification, 20×), 20 µm (magnification, 63×)].

To investigate the effects of CS as a systemic inducer of inflammation, *in vivo* and *in vitro* studies were performed. First, C57BL/6 mice were exposed to CS for 2 × 30 min each day over 2 months. The test animals developed emphysema-like tissue damage (Figure 1A) that correlated with decreased epithelial cell number (Figure 1B). Epithelial cells were also separated from the CD45^+^ leukocyte population (Figure 1B). Both the epithelial cell enriched and the CD45^+^ cell populations had elevated Wnt5a mRNA levels (Figure 1C). Wnt5a protein expression levels were also increased (Figures 1D,E). Different isoforms of CD45 are expressed in many leukocyte populations including T and B lymphocytes and antigen-presenting cells, no effect of these immune cells, except macrophages, were investigated further. To test whether CSE can trigger Wnt5a up-regulation in human lung tissues, *in vitro* human lung aggregate cultures were used. The aggregate cultures contained SAEC, mesenchyme (NHLF), and endothelial cells (HMVEC-L) in the presence or absence of human blood monocyte-derived macrophages and were treated with CSE for a maximum of 48 h. The CSE treated aggregates have shown increased levels of Wnt5a both at mRNA and protein levels but only if monocytes were present in the aggregate tissues (Figures 2A–D) (45, 46).

### Increased Inflammatory Cytokine Production in the Lung After CS Exposure Is Associated With the Presence of Macrophages

**Figure 3:**
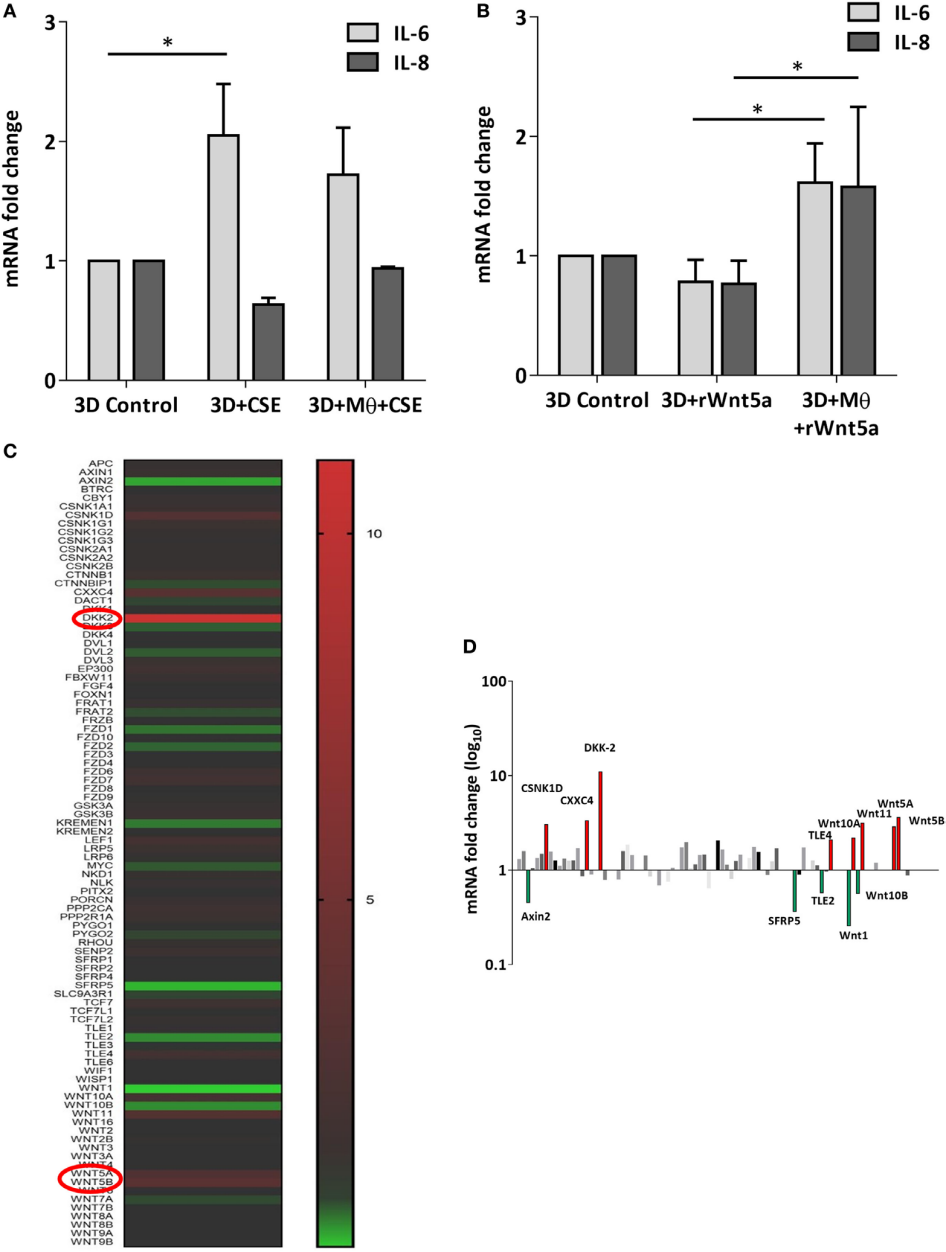
Inflammatory cytokine and Wnt mRNA levels in primary human 3D lung aggregate cultures and human macrophages (M) after cigarette smoke extracts (CSE) exposure. **(A)** IL-6 and IL-8 inflammatory cytokine mRNA levels in control and CSE exposed (48 h) lung aggregate cultures, containing and not containing M. **(B)** IL-6 and IL-8 inflammatory cytokine mRNA levels in control and rWnt5a (1 µg/ml) treated lung aggregate cultures, containing and not containing M. **(C)** Taqman array heat map analysis of pooled (*n* = 4) cDNA of human M compared with control. M were treated with CSE for 3 h. **(D)** Changes in mRNA levels of Wnt signaling pathway genes measured by Taqman Array analysis using pooled (*n* = 4) cDNA samples of human M compared with control.

**Figure 4:**
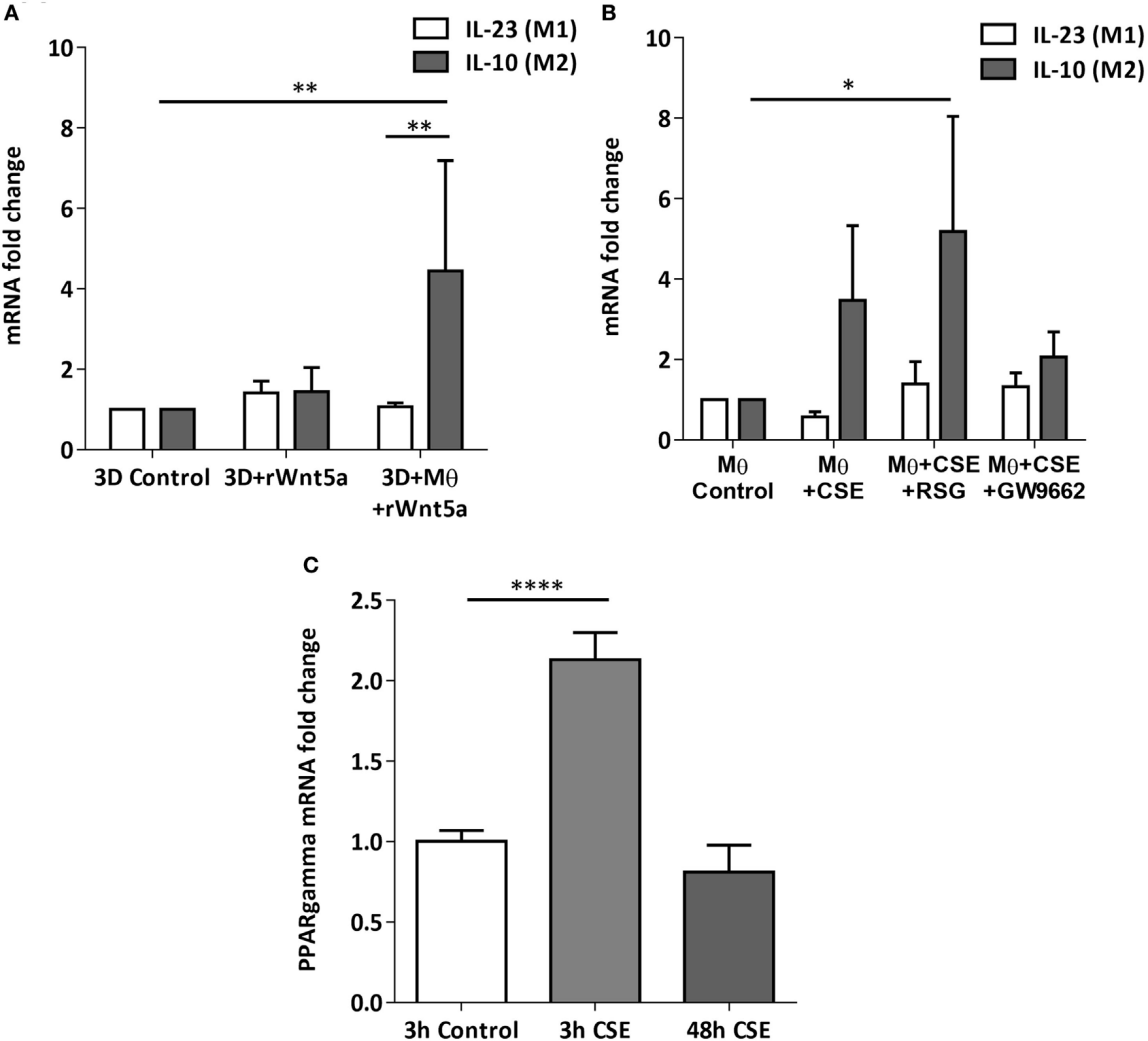
Changes of peroxisome proliferator-activated receptor (PPAR) gamma mRNA levels in M1 and M2 M type differentiation in primary human 3D lung aggregate cultures and M monocultures. **(A)** qRT-PCR analysis of M1 (differentiation marker IL-23) and M2 (differentiation marker IL-10) after human rWnt5a (1 µg/ml) treatment for 48 h. **(B)** Effects of cigarette smoke extracts (CSE), PPAR gamma agonist (RSG), and antagonist (GW9662) treatment on expression of M1 (IL-23) and M2 (IL-10) differentiation markers. CSE treatment (48 h) and also CSE + RSG (10 µM) and CSE + GW9662 (10 µM) treatment on human M. **(C)** PPAR gamma mRNA levels in CSE treated human macrophage cultures after 3 and 48 h of CSE treatment compared with controls.

After the recruitment of immune cells to help in eliminating foreign materials and to aid tissue repair after damage or infection, immune reactions are resolved and the tissue homeostasis is normalized. Macrophages are involved in self-limiting, regulated resolution of inflammatory processes at different levels. Tissue-resident macrophages and activated monocytes can adopt different activation status, and although the process is still not fully characterized, M1 and M2 type polarization can provide an outline of how macrophage activity can change during inflammation. To test the effects of CS on the human lung, aggregate cultures of primary human lung cell types were exposed to CSE for 48 h and pro-inflammatory cytokine gene expressions were measured. Both in the presence or absence of macrophages, significant increase of IL6 peptide levels were detected compared to untreated controls (Figure 3A). If the human lung aggregate cultures were exposed to rhWnt5a (1 µg/ml, for 48 h) then both IL-6 and IL-8 peptide levels increased significantly in the monocyte containing tissue cultures indicating a sequential activation of pro-inflammatory cytokine and pro-inflammatory Wnt5a production (Figure 3B). To attempt to dissect such a dynamic process, blood monocytes were exposed to CSE, then, Wnt signaling TaqMan Array was performed (Figures 3C,D). Compared to untreated controls, up-regulation of characteristic non-canonical Wnt signaling molecules were detected, including Wnt5a, Wnt5b, Wnt11 as ligands and an inhibitor of the canonical Wnt pathway, Dickkopf 2 (DKK-2) (Figures 3C,D). In addition to specific molecules of the Wnt pathway, increased transcription of CXXC4—a member of the zinc finger domain-containing protein family—was detected. Up-regulation of CXXC4 that functions as an antagonist of the canonical Wnt/integrated signaling pathway indicates active suppression of the canonical Wnt pathway. Furthermore, increased mRNA levels of CSNKD1 a casein kinase I gene family member, transducin-like enhancer of split 4 (TLE4), and Wnt10a were also detected. While CSNKD1 is an essential serine/threonine-protein kinase and regulator of diverse cellular growth and survival; TLE4 is a transcriptional corepressor. Overexpression of the Wnt10a gene may be of particular interest as Wnt10a can play key roles in carcinogenesis through activation of the Wnt-beta-catenin-TCF signaling pathway. Simultaneously, reduced transcription of Axin2 that plays an important role in the regulation of beta-catenin stability and a characteristic canonical Wnt ligand, Wnt1, were down-regulated. Reduced transcription of TLE2 and secreted frizzled-related protein 5 was also observed, demonstrating deregulation of Wnt signaling in macrophages as a direct effect of CS exposure. Deregulation of either the canonical or the non-canonical Wnt signaling results in modification of PPAR gamma expression and activity (31). To investigate how such changes in the Wnt signaling pathway can affect the dynamic polarization of macrophages, pro-inflammatory cytokines characteristic to the pro-inflammatory M1, and the anti-inflammatory but pro-tumorigenic M2 phenotypes (47) were measured in association with PPAR gamma expression and activity. To study how PPAR gamma is involved in the CS induced Wnt5a up-regulation as well as consequent pro-inflammatory cytokine production, human lung aggregate cultures were exposed to Wnt5a and CSE in the presence or absence of PPAR gamma agonist, rosiglitazone (RSG), and inhibitor (GW9662) (Figures 4A,B). The M1 phenotype is mostly characterized by pro-inflammatory cytokines IL-6, IL-8, IL-23, and IL-1β, while M2 by anti-inflammatory cytokines IL-10 and TGF-beta (34). In the studied time frame, inhibition of the anti-inflammatory PPAR gamma by GW9662 reduced the gene expression of IL-10—the characteristic cytokine marker of the anti-inflammatory M2 phenotype (Figure 4B). Cytokine production, however, is a dynamic process. In order to investigate it, human blood monocyte-derived macrophages were exposed to CSE for two different time points 3 and 48 h, then PPAR gamma transcription was measured. Interestingly, at the 3 h time point the anti-inflammatory PPAR gamma transcription significantly increased, but by 48 h the transcript level reduced to control levels (Figure 4C) indicating that expression of the anti-inflammatory PPAR gamma would not keep up with the prolonged inflammatory stimuli.

### Chronic CS Exposure Increases Wnt5a Protein Levels in EV-s of COPD Patients’ Sera

**Figure 5:**
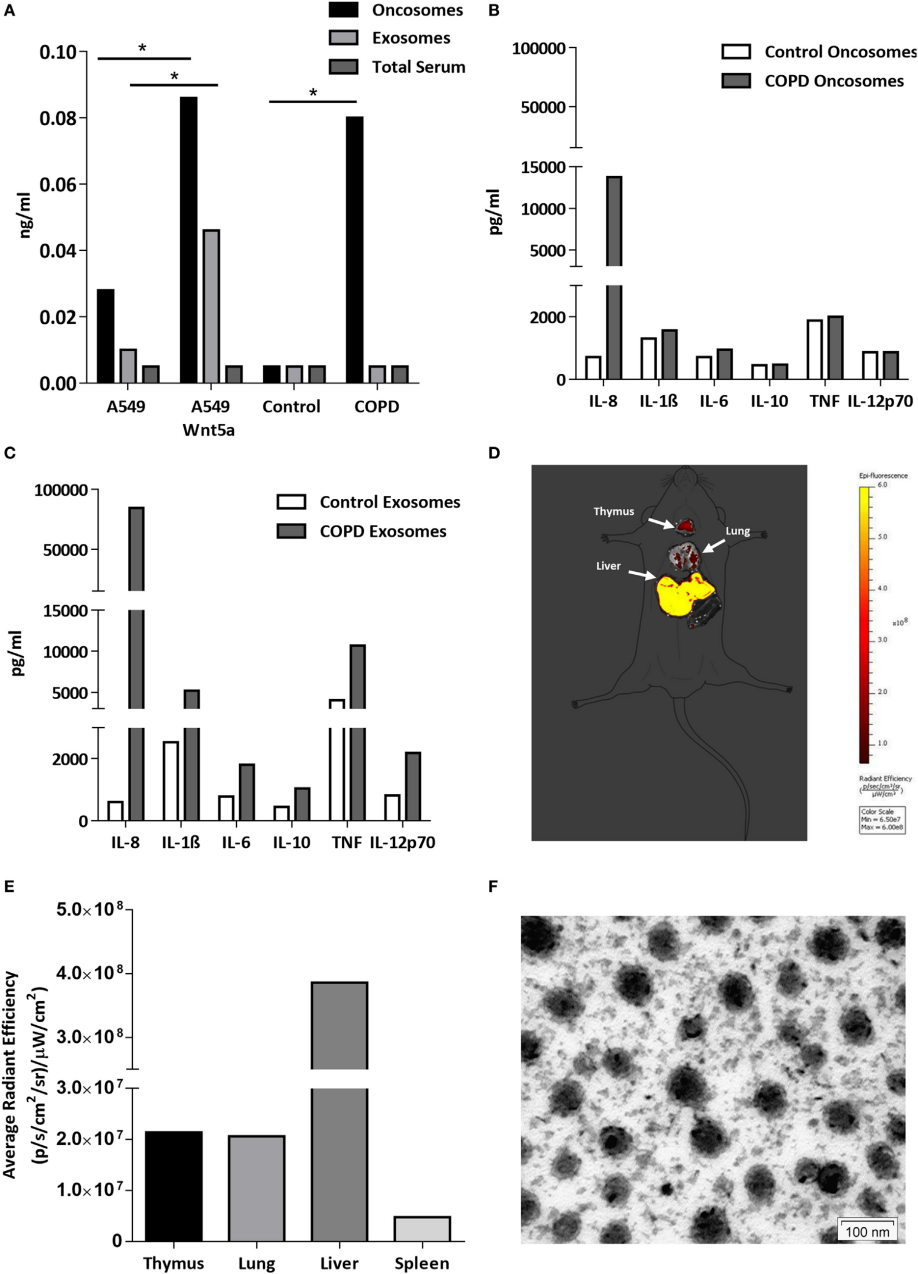
Wnt5a and proinflammatory cytokine levels in extracellular vesicles (EV-s). **(A)** Wnt5a protein levels were measured in EV-s in sera of healthy donors and chronic obstructive pulmonary disease (COPD) patients as well as EV-s of A549 and Wnt5a-A549 cell lines as controls using a Wnt5a-specific enzyme-linked immunosorbent assay kit. **(B)** Inflammatory cytokine levels were measured in pooled megavesicles/oncosome samples of five healthy and five COPD patients isolated from human sera measured by cytometric bead array Flex kit using flow cytometry. **(C)** Inflammatory cytokine proteins were detected in pooled microvesicle/exosome samples of five healthy and five COPD patients isolated from human sera. **(D)** Fluorescent staining (Dil) labeled Wnt5a-containing EV-s are detectable in various organs (representative image of *n* = 3). **(E)** Fluorescence intensity of Wnt5a containing EV-s in various organs (representative data of *n* = 3). **(F)** Electron microscopic image of purified EV-s (microvesicle/exosome population) purified from the Wnt5a-A549 cell line. Scale bar is 100 nm.

The lipo-glycoprotein Wnt ligands are highly hydrophobic (48), therefore, their transport in aqueous body fluids is facilitated by EV-s (49). EV-s (both megasomes/oncosomes or microsomes/exosomes) are now widely accepted as a novel route of cellular communication that can influence the long-range Wnt signal gradients (50, 51). Based on the above, we hypothesized that Wnt5a and low serum levels of pro-inflammatory cytokines can be measured in EV-s isolated from human sera (52, 53). If EV-s can travel from the organ of origin and reach any cells and organs of the human body safely delivering their contents, such transport system would explain the systemic nature of COPD. To test our theory, the larger megavesicles/oncosomes and the smaller microvesicles/exosomes were isolated from sera of COPD patients as well as age-matched healthy controls. The non-small cell carcinoma cell line A549 and its engineered Wnt5a overexpressing sister cell line Wnt5a-A549 were used as positive controls. Wnt5a was measured by ELISA (Figure 5A), whereas pro-inflammatory cytokines were measured by a bead assay using flow cytometry (Figures 5B,C). While Wnt5a was not detectable in sera of neither COPD patients nor age-matched healthy volunteers, megavesicles/oncosomes isolated from sera of COPD patients contained similar levels of Wnt5a as measured in the Wnt5a overexpressing Wnt5a-A549 cell line (Figure 5A). Similarly, while many pro-inflammatory cytokines remained undetectable in pure human sera (data not shown), exosomes contained high levels of such cytokines (Figure 5C) with an extremely high level detected for IL-8 (Figure 5C).

To investigate whether these concentrated “cellular messages” can reach other organs, extracellular vesicles were isolated from the supernatant of the Wnt5a-A549 cell line and the lipid bilayer was stained with a DiI fluorescent dye to be injected into the tail veins of Balb/c mice. Mice were sacrificed after 20 h and the organs were removed to detect fluorescence. The experiments provided evidence that inflammatory cytokine containing microvesicles/exosomes can reach and become concentrated in the liver, lungs, spleen, and thymus of test animals (Figures 5D–F).

## Discussion

**Figure 6:**
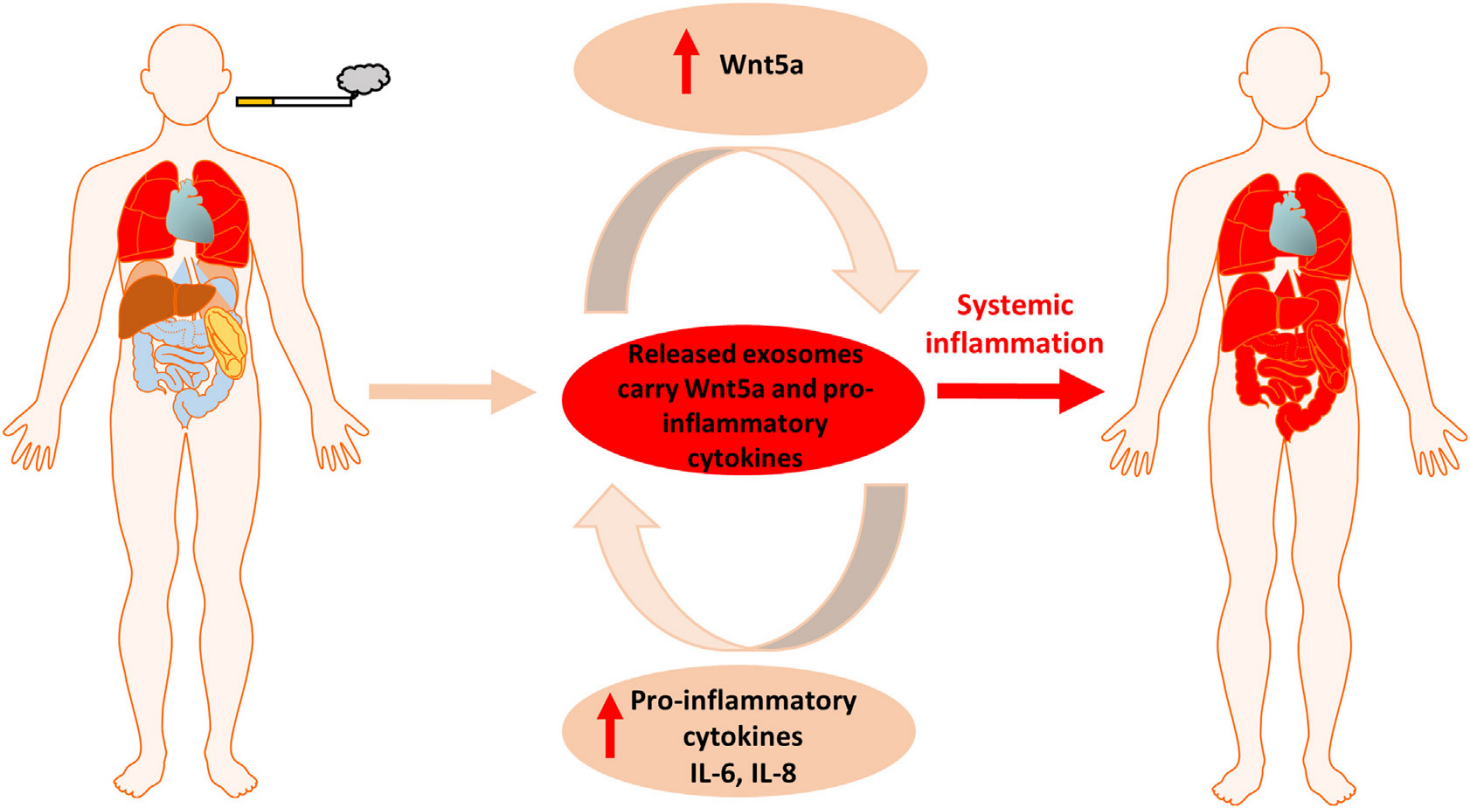
Summary figure of cigarette smoke induced local inflammation becoming systemic by the contribution of Wnt5a and inflammatory cytokines.

Cigarette smoke induced chronic lung inflammation, PPAR gamma activity, Wnt signaling deregulation and increased overall risk of carcinogenesis are all interconnected (54, 55). Our report is the first to shed light on a potential mechanism. We have shown that CS induces the expression of molecules of the non-canonical Wnt signaling pathway that simultaneously suppress the canonical Wnt pathway. This observation is significant as the canonical Wnt signaling is essential to maintain stem cell niches in the lung (56). In fact, CS creates a tissue microenvironment that is similar to the ones observed during aging (43). Similarly to smokers, the aging microenvironment is associated with a significant decrease of the EpCAM^+^ epithelial cell layer that correlates with emphysema and decreased gas exchange surface (43). In parallel, inflammatory environment is present marked by the increased number of the CD45^+^ leukocyte population of immune cells in the tissue of the lung and up-regulated non-canonical Wnt signaling (43). While the lung is young and healthy, inflammatory respiratory diseases are kept in check by dynamic polarization of macrophages into the anti-inflammatory M2 subsets that suppress the pro-inflammatory M1 macrophage phenotype (57). PPAR gamma plays an important and similarly dynamic anti-inflammatory role by inducing M2 type polarization (39, 58). Prolonged exposure to CS, however, re-enforces the “aging” process by reducing PPAR gamma expression. Repeated exposure to CS also alters the lung tissue homeostasis and the Wnt microenvironment that closely interacts with PPAR gamma (59). Although the precise molecular regulation remains largely unclear, PPAR gamma (60, 61) and the Wnt signaling pathways (62) have been suspected to somehow regulate the systemic nature of COPD. The non-canonical Wnt5a that is associated with aging (43), carcinogenesis (30), as well as with activation of pro-inflammatory processes during M1 phenotype macrophage differentiation and pro-inflammatory cytokine production (63–65) was up-regulated upon CS exposure and was found in megavesicles/oncosomes of COPD patients. Wnt5a can also suppress levels of the anti-inflammatory PPAR gamma *via* increasing miR27b that binds to the PPAR gamma promoter and blocks its transcription (31). As both pro-inflammatory cytokines and Wnt5a can be wrapped and protected by the lipid bilayer of EV-s then not just the inflammation can become systemic but also the inhibition of the anti-inflammatory activity of PPAR gamma (Figure 6). Although previous studies hypothesized that PPAR gamma agonist treatments can modulate and suppress inflammation (35, 66) or even reverse CS-induced emphysema (37), our experiments have highlighted the difficulty of achieving such anti-inflammatory therapy targeting solely PPAR gamma. Furthermore, as PPAR gamma agonists can promote M2 phenotype differentiation of macrophages (39), such PPAR gamma agonist therapy can even lead to unintended promotion of carcinogenesis.

In summary, in the present study we demonstrated that during cigarette smoking the inflammation inducer and PPAR gamma inhibitor Wnt5a as well as pro-inflammatory cytokines can be carried to every organ in the human body in various types of extracellular vesicles triggering systemic inflammation and making COPD a complex disease that is hard to control. Further studies are needed to investigate how to utilize our discovery in identification of differential diagnostic and prognostic markers as well as application of extracellular vesicles in targeted therapy.

## Ethics Statement

Animal subjects: The *in vivo* animal studies were approved by the Ethics Committee on Animal Research of Pecs University according to the Ethical Codex of Animal Experiments (license no. BA 02/200-6-2001). The protocol was approved by the Ethics Committee on Animal Research of Pecs University. Human subjects: This study was carried out in accordance with the recommendations of Ethics Committee on Human Research, Ethical Committee of the University of Pecs (ETT 6444/2016). The protocol was approved by the Ethical Committee of the University of Pecs. All subjects gave written informed consent in accordance with the Declaration of Helsinki.

## Author Contributions

JP, DF, KK, and ZH contributed conception and design of the study. DF, JR, and JK performed experimental work. DF and JR performed the statistical analysis. IR and VS designed and selected patients, collected sera, and advised on clinical aspects. JP wrote the first draft of the manuscript. DF wrote sections of the manuscript. All authors contributed to manuscript revision, read and approved the submitted version.

## Conflict of Interest Statement

The authors declare that the research was conducted in the absence of any commercial or financial relationships that could be construed as a potential conflict of interest.
