# Supplementary material for: Cigarette Smoke-Induced Pulmonary Inflammation Becomes Systemic by Circulating Extracellular Vesicles Containing Wnt5a and Inflammatory Cytokines
Source: Front Immunol. 2018 Jul 25;9:1724. doi: 10.3389/fimmu.2018.01724 (PMC6068321; doi:10.3389/fimmu.2018.01724)
Supplement: Supplementary file 1 [file data_sheet_1.docx]

**Supplementary data**

**Supplementary figure 1. qRT-PCR analysis of selected genes identified as up-regulated by Taqman Array in human macrophages.** Changes in mRNA levels of selected Wnt ligand genes were measured in individual samples (n=4).
